# Endogenous pH 6.0 β-Galactosidase Activity Is Linked to Neuronal Differentiation in the Olfactory Epithelium

**DOI:** 10.3390/cells11020298

**Published:** 2022-01-16

**Authors:** José Antonio de Mera-Rodríguez, Guadalupe Álvarez-Hernán, Yolanda Gañán, Ana Santos-Almeida, Gervasio Martín-Partido, Joaquín Rodríguez-León, Javier Francisco-Morcillo

**Affiliations:** 1 Área de Biología Celular, Departamento de Anatomía, Biología Celular y Zoología, Facultad de Ciencias, Universidad de Extremadura, 06006 Badajoz, Spain; merarodja@unex.es (J.A.d.M.-R.); galvarezt@unex.es (G.Á.-H.); santosana1991@gmail.com (A.S.-A.); gmartin@unex.es (G.M.-P.); 2 Área de Anatomía y Embriología Humana, Departamento de Anatomía, Biología Celular y Zoología, Facultad de Medicina y Ciencias de la Salud, Universidad de Extremadura, 06006 Badajoz, Spain; yolandag@unex.es

**Keywords:** β-Galactosidase, cell differentiation, histochemistry, immunohistochemistry, mouse, olfactory epithelium, senescence

## Abstract

The histochemical detection of β-galactosidase enzymatic activity at pH 6.0 (β-gal-pH6) is a widely used biomarker of cellular senescence in aging tissues. This histochemical assay also detects the presence of programmed cell senescence during specific time windows in degenerating structures of vertebrate embryos. However, it has recently been shown that this enzymatic activity is also enhanced in subpopulations of differentiating neurons in the developing central nervous system in vertebrates. The present study addressed the histochemical detection of β-gal-pH6 enzymatic activity in the developing postnatal olfactory epithelium in the mouse. This activity was detected in the intermediate layer of the olfactory epithelium. As development progressed, the band of β-gal-pH6 labeling in this layer increased in width. Immunohistochemistry and lectin histochemistry showed the β-gal-pH6 staining to be strongly correlated with the immunolabeling of the olfactory marker protein (OMP) that identifies mature olfactory sensory neurons. The cell somata of a subpopulation of differentiated olfactory neurons that were recognized with the Dolichos biflorus agglutinin (DBA) were always located inside this band of β-gal-pH6 staining. However, the β-gal-pH6 histochemical signal was always absent from the apical region where the cytokeratin-8 positive supporting cells were located. Furthermore, no β-gal-pH6 staining was found in the basal region of the olfactory epithelium where PCNA/pHisH3 immunoreactive proliferating progenitor cells, GAP43 positive immature neurons, and cytokeratin-5 positive horizontal basal cells were located. Therefore, β-gal-pH6 seems to be linked to neuronal differentiation and cannot be regarded as a biomarker of cellular senescence during olfactory epithelium development in mice.

## 1. Introduction

The most distinctive measurable feature of cellular senescence in vivo and in vitro is the presence of a specific β-galactosidase enzymatic activity at pH 6.0 (β-gal-pH6, also known as “senescence-associated β-galactosidase”), different from that normally observed at pH 4.5 within lysosomes [1,2]. This histochemical enzymatic indicator has been used to study certain aspects of cell and organism aging and several diseases, including cancers [3,4]. In addition to these pathological and non-pathological conditions in adult organisms, vertebrate embryos stained with β-gal-pH6 exhibit intense labeling in specific anatomical structures and locations [5,6,7,8,9,10,11,12]. Embryonic senescent cells have been reported to be non-proliferative and subject to clearance from tissues after programmed cell death [6,13]. However, some authors have concluded that β-gal-pH6 activity is enhanced under a variety of cellular physiological conditions, even in embryonic tissues. Endogenous histochemical staining is detected in the mouse visceral endoderm at early stages of development [14], in embryonic cardiomyocytes, skeletal muscle myofibers, osteoblasts, and cochlear hair cells [15,16], and embryonic macrophages [12]. Li et al., 2018 [17] establish a link between the concepts of cellular senescence and cell differentiation, showing that, at more advanced stages of development, β-gal-pH6 positive cells located in the apical ectodermal ridge of the limb re-enter the cell cycle, proliferate, and differentiate in epithelial cells. In the case of the nervous system, intense β-gal-pH6 staining has been detected in different subsets of neurons in very young mice (1–3 months old) [18,19,20,21]. Furthermore, neurons of the trigeminal ganglion and cerebellar Purkinje cells also exhibit enhanced expression of β-gal-pH6 during the first postnatal days in the mouse [12]. There is also enhanced β-gal-pH6 activity in motoneurons in the mouse embryonic spinal cord [22] and in the first differentiating neurons detected in the embryonic retina, suggesting that it has some link with neuronal differentiation [12,23,24].

The olfactory epithelium is an interesting model for studying ontogenetic cell death and developmental and adult neurogenesis [25]. In this pseudostratified epithelium, cell proliferation, cell differentiation, and cell death are highly regulated processes that begin during embryonic development and continue throughout adult life, including in mammals [26,27]. Continuous mitotic division of globose basal cells produces new olfactory neuron precursors that migrate apically, differentiating into mature olfactory neurons [28,29]. In mammals, a large proportion of the new olfactory neurons generated usually die by apoptosis under physiological conditions in the developing and adult olfactory epithelium [27,30,31,32]. Most of these studies clearly demonstrate age-related changes in cell production, neuronal differentiation’s temporal course, and the magnitude and distribution of cell death in the olfactory epithelium [27].

All of these findings led us to think that the developing postnatal olfactory epithelium might well constitute an excellent model for studying whether β-gal-pH6 is linked with cell differentiation and/or apoptosis. We, therefore, set out to (a) investigate the presence of enhanced β-gal-pH6 during the development of the mouse olfactory epithelium during early postnatal life and (b) identify the populations of cells that exhibit high levels of β-gal-pH6 staining.

## 2. Materials and Methods

### 2.1. Animal Model and Tissue Processing

A total of 21 postnatal Swiss/ICR albino mice of different ages were used in the present study. All the animal studies were performed in accordance with the National and European legislation (Spanish Royal Decree RD53/2013 and EU Directive 86/609/CEE as modified by 2003/65/CE, respectively). Experimental protocols were approved by the Bioethics Committee for Animal Experimentation of the University of Extremadura (Protocol code 266/2019, 29 July 2020). The animals studied ranged from the day of birth (postnatal day 0, P0) to P60. They were deep anesthetized and fixed by means of intracardiac perfusion with 4% paraformaldehyde (PFA 4%) in 0.1 M PBS solution (pH 7.4) at 4 °C, and then decapitated. The entire heads of the animals were post-fixed by immersion in the same fixative at 4 °C overnight. The skin was withdrawn, and the heads were immersed in saturated decalcification solution 20% EDTA in distilled water (pH 7.4) with shaking at 4 °C for one week until the bone was soft and pliable. The solution was changed every two days.

Heads were cryoprotected in 15% sucrose solution in PBS and embedded in 15% gelatin 15% sucrose solution in the same buffer. The blocks were frozen for 5 min in 2-propanol cooled to −70 °C by dry ice and then stored at −80 °C. Cryostat serial coronal, horizontal, and sagittal sections were obtained, mounted on SuperFrost-Plus slides (Menzel-Glaser, Germany), air-dried, and stored at −20 °C until use.

### 2.2. Detection of β-Galactosidase Activity

Cryosections were incubated at 37.5 °C with 350 μL of the chromogenic β-gal substrate X-gal (5-bromo-4-chloro-3-indolyl-β-D-galactopyranoside, also called BCIG) in PBS-MgCl2 at pH 6.0 for 24 h, as has been described for cryostat sections [19]. β-gal-pH6 positive cells develop a blue-green precipitate. The X-gal solution was removed, and the sections were washed with PBS-MgCl2 acid buffer for 10 min. After the histochemical reaction, sections were counterstained with DAPI (Sigma-Aldrich, Madrid, Spain, Ref. D9542) for 10 min at room temperature in darkness. Then the slides were washed in PBS and mounted with Mowiol (polyvinyl alcohol 40–88, Fluka, Madrid, Spain, Ref. 81386) for observation. The fixation conditions had to be kept as mild as possible since prolonged incubation times or severe conditions can destroy β-gal-pH6 activity. For double-labeling purposes, the β-gal-pH6 histochemistry was performed first, followed by the immunohistochemical/lectin histochemical procedures (see below).

### 2.3. Immunohistochemistry

Working solutions and sources of primary antibodies used in the present study included the following:

1. Goat anti-olfactory-marker-protein (OMP) polyclonal antibody, used at 1:100 (Wako Chemicals, Neuss, Germany; Ref. 544-10001/IUP1001), which identifies mature olfactory sensory neurons [33].
2. Mouse anti-cytokeratin 8 (CyK8) monoclonal antibody, used at 1:50 (Novusbio, Centennial, CO, USA, Ref. NB120-9287), which stains supporting cells in the olfactory epithelium [34].
3. Mouse anti-proliferating cell nuclear antigen (PCNA; PC10) monoclonal antibody, used at 1:100 (Santa Cruz Biotechnology, Heidelberg, Germany; Ref. sc-56), which identifies proliferating cells in the olfactory epithelium in the fish olfactory epithelium [35] and in other sensory systems [24,36,37].
4. Rabbit anti-cytokeratin 5 (CyK5) polyclonal antibody, used at 1:200 (BioLegend, San Diego, CA, USA; Ref. 905501/D14FF0122), which identifies horizontal basal cells [38,39].
5. Rabbit anti-phospho-histone H3 (pHisH3) polyclonal antibody, used at 1:100 (Millipore, Ref. 06-570), a marker for mitotic cells in the developing olfactory system [40] and in other sensory systems [41].
6. Rabbit anti-Growth associated protein-43 (GAP43) polyclonal antibody, used at 1:200 (Millipore, Darmstadt, Germany; Ref. AB5220), a marker of immature neurons in the olfactory system [38].

**Figure 1:**
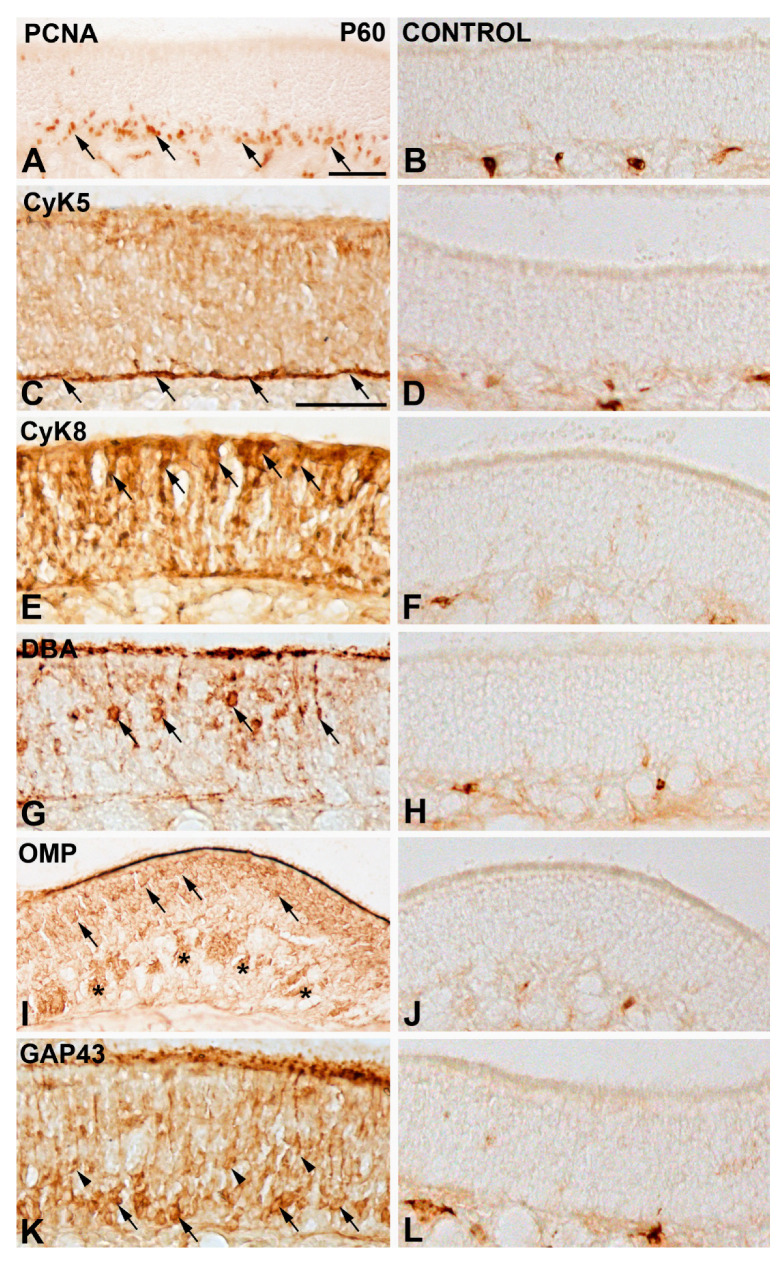
Patterns of staining for the different antibodies and the lectin used in the present study in the P60 mouse olfactory epithelium (**A**,**C**,**E**,**G**,**I**,**K**). Negative controls (**B**,**D**,**F**,**H**,**L**) included the replacement of primary antibodies by PBS. PCNA immunoreactive nuclei were observed in the basal olfactory epithelium (arrows in (**A**)). Intense immunoreactivity against CyK5 was confined to the layer where the horizontal basal cells were located (arrows in (**C**)). Strong immunoreactivity against CyK8 was detected in the cell somata located in the apical surface of the olfactory epithelium (arrows in (**E**)). DBA-stained olfactory neurons appeared in the middle part of the olfactory epithelium (arrows in (**G**)). Mature olfactory neurons expressing OMP (arrows in (**I**)). Anti-OMP staining was also localized in nerve bundles dispersed throughout the lamina propria (asterisks). (**K**) GAP43-immunoreactive immature neurons were mainly located in the basal region of the olfactory epithelium (arrows in (**K**)) and in sparse cell somata located in the central region (arrowheads in (**K**) Scale bars: 100 µm in (**A**–**F**); 50 µm in (**G**).

To test these antibodies’ efficacy, we have conducted immunohistochemical analyses on cryosections of the mouse olfactory epithelium. These antibodies showed robust immunolabelling for PCNA, CyK5, CyK8, OMP, and GAP43 in tissue from P60 specimens (Figure 1A,C,E,I,K, respectively). As negative controls, the primary antibodies were omitted from the reaction (Figure 1B,D,F,J,L, respectively). Secondary antibodies used in the present study included Alexa Fluor 488 goat anti-mouse IgG antibody (Molecular Probes, Madrid, Spain; Ref.A11029), Alexa Fluor 594 goat anti-rabbit IgG antibody (Molecular Probes, A11037), Alexa Fluor 568 donkey anti-goat IgG antibody (Molecular Probes, AB_2534104), goat anti-mouse IgG Biotin-conjugated (Invitrogen, Madrid, Spain; Ref. 62-6540), and rabbit anti-goat IgG Biotin-conjugated (Sigma-Aldrich, Madrid, Spain, Ref. B 7014). The dilution used for all the secondary antibodies was 1:200.

Single and double immunohistochemical techniques were performed as described by Bejarano-Escobar et al. (2011, 2013) [42,43]. After immunofluorescence, the slides were washed in PBS, counterstained with DAPI, and mounted with Mowiol. Some cryostat sections were washed several times in 0.05% Triton X-100 in PBS (PBS-T) and treated with 3% hydrogen peroxide in PBS solution for 45 min. After rinsing with PBS-T, sections were pre-blocked in 0.2% gelatin, 0.25% Triton X-100, Lys 0.1 M in PBS (PBS-G-T-L) for 2 h and incubated overnight with primary antibody solution in a humidified chamber at room temperature (RT). The slides were washed three times in PBS-G-T-L and incubated with secondary antibody solution for 4 h at RT. After rinsing, the slides were treated with a solution of 1:200 diluted ExtrAvidin-peroxidase (Sigma, SA-5004) for 2 h. Following another rinse, 0.05% 3,3′-diaminobenzidine tetrahydrochloride (DAB) and a 0.02% hydrogen peroxide solution were added. Finally, the sections were mounted in Eukitt (Kindler, Freiburg, Germany) and coverslipped for observation.

### 2.4. Dolichos Biflorus Agglutinin (DBA) Histochemistry

We used a lectin obtained from *Dolichos biflorus* with an affinity for N-acetyl-D-galactosamine sugar residues. This permits the identification of a subpopulation of olfactory neurons in the mouse [44]. Cryostat sections were washed several times in 0.05% Triton X-100 in PBS (PBS-T) and treated with 3% hydrogen peroxide in PBS solution for 45 min. After rinsing twice in PBS and once in PBS-T for 10 min, sections were incubated with biotinylated DBA (Vector Laboratories, San Cugat del Vallés, Barcelona, Spain; B-1035-5) at a 5 mg/mL concentration in PBS-T overnight at room temperature. The slides were rinsed twice in PBS for 15 min and incubated with a solution of 1:200 diluted ExtrAvidin-peroxidase or with ExtrAvidin-fluorescein isothiocyanate (FITC) conjugated (Sigma, E2761) for 2 h at RT. After rinsing twice in PBS for 15 min, the peroxidase reaction product was visualized with 0.05% 3.30-diaminobenzidine tetrahydrochloride (DAB) and 0.025% hydrogen peroxide in PBS for 10 min at room temperature. The sections were then mounted with Mowiol. Strong staining was detected in cryosections with DBA (Figure 1G). Negative controls for the lectin histochemistry included the omission of primary reagent (biotinylated lectin) and only the DAB solution (Figure 1H).

### 2.5. TUNEL Technique

Because most cell death processes during development occur by apoptosis [45], more recent approaches to map areas of cell death are based on the detection of DNA fragmentation on cryosections using the TUNEL (terminal deoxynucleotidyl transferase (TdT)-mediated deoxyuridinetriphosphate nick end-labeling) technique [27] with the in situ Cell Death Detection Kit, POD (Roche Molecular Biochemicals, Mannheim, Germany). The TUNEL technique was performed as described in Francisco-Morcillo et al. (2004) [46]. No stained nuclei were observed in control sections in which the enzyme TdT had been omitted from the reaction solution.

### 2.6. Image Acquisition and Processing

Digital images of the developing mouse olfactory epithelium sections were observed under a Nikon Eclipse-80i photo-microscope equipped with bright-field and fluorescence microscopy capacities and photographed using an ultra-high-definition Nikon DXM 1200F. The assembly of the resulting images was done with Adobe Photoshop CS4.

**Figure 2:**
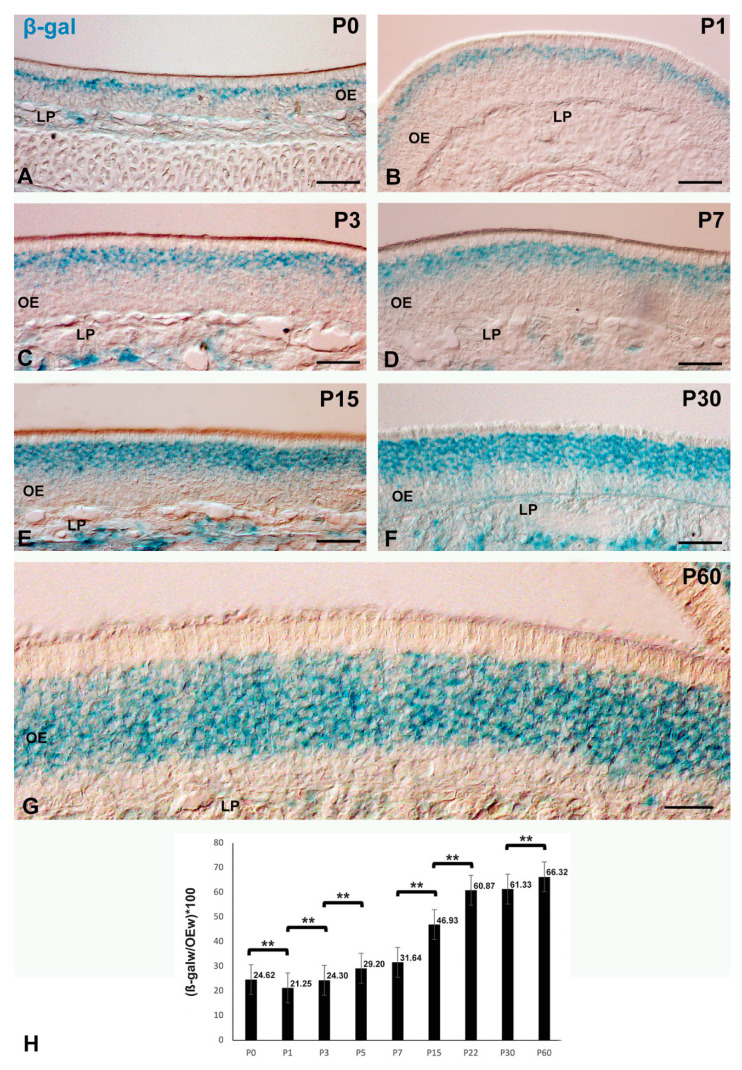
The presence of β-gal-pH6 histochemical staining in the developing olfactory epithelium of P0 (**A**), P1 (**B**), P3 (**C**), P7 (**D**), P15 (**E**), P30 (**F**), and P60 (**G**) mice. β-gal-pH6 staining was restricted to the intermediate zone of the olfactory epithelium. (**H**) Quantitative analysis of the relationship between the width of the band of β-gal-pH6 staining and the thickness of the olfactory epithelium (OEw) during postnatal mouse development. Data are expressed as mean ± SEM. Statistical significance is indicated by asterisks (** *p* < 0.01). The width of the β-gal-pH6 staining band increased progressively with age. LP, lamina propria; OE, olfactory epithelium. Scale bars: 100 µm in (**A**–**F**); 50 µm in (**G**).

The widths of the β-gal-pH6 labeling band and of the olfactory epithelium were quantified using the ImageJ (http://rsb.info.nih.gov/ij/ accessed on 17 December 2021, software package (Version 1.38). A total of 20 randomly selected images per specimen (three specimens per developmental stage) were analyzed for this purpose. Three measurements per section were made in the apical-basal axis of the epithelium at different levels of the nasal septum and turbinates and different regions in the anterior-posterior extent. Each width measurement of the β-gal-pH6 labeling band was divided by the width of the olfactory epithelium and multiplied by 100 to get the percentage increase in the labeling width. Statistical analyses were performed using a two-sample t-test assuming equal variances. Differences between groups were considered as significant when *p* < 0.05 (Figure 2H).

## 3. Results

### Histochemistry of β-gal-pH6 in the Developing Postnatal Mouse Olfactory Epithelium

Figure 2 illustrates the staining pattern of β-gal-pH6 in the mouse olfactory epithelium during the first two months of postnatal life. The β-gal-pH6 staining was observed in cryosections of the olfactory mucosa of all ages examined (Figure 2). The intense β-gal-pH6 activity was mainly detected in a thin band located in the intermediate zone of the olfactory neuroepithelium on the day of birth (P0) (Figure 2A). During the first week of life the band of staining increased in size in this same region (Figure 2B–D,H). This increase in the width of the β-gal-pH6 labeling band was more evident (Figure 2H) in 15- (Figure 2E), 30- (Figure 2F), and 60-day-old (Figure 2G) mice.

**Figure 3:**
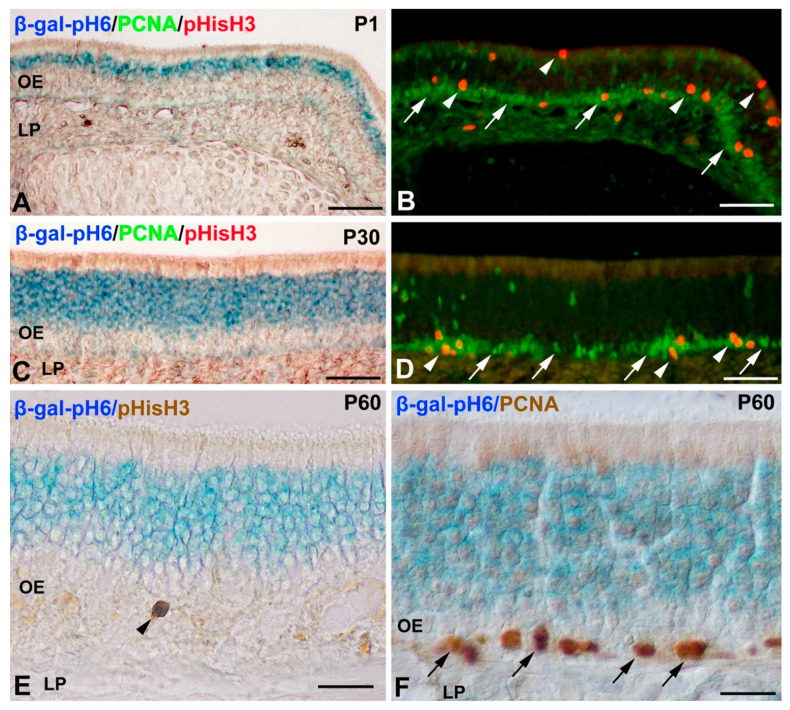
Relationship between β-gal-pH6 histochemical staining and markers of proliferation in the olfactory epithelium of mice of different postnatal ages. Cryosections were triply labeled with β-gal-pH6 histochemistry and PCNA/pHisH3 antibodies (**A**–**D**) or doubly labeled with β-gal-pH6 histochemistry and antibodies against pHisH3 (**E**) or antibodies against PCNA (**F**). (**A**,**B**) At P0, PCNA immunoreactive nuclei (green, arrows) and pHisH3 immunolabeled mitoses (red, arrowheads) were mainly located in the basal and apical regions of the olfactory epithelium. A band of β-gal-pH6 staining was found in the epithelium region where the olfactory neurons were located. (**C**–**F**) At P30 and P60, a wide band of intense β-gal-pH6 labeling was found in the intermediate layer of the olfactory epithelium. PCNA immunoreactive nuclei (arrows) and pHisH3 immunolabeled mitoses (arrowheads) were mainly located in the basal region of the epithelium. Some PCNA-immunolabeled nuclei were found at different depths of the epithelium (**D**). LP, lamina propria; OE, olfactory epithelium. Scale bars: 100 µm in (**A**–**D**); 50 µm in (**E**,**F**).

Next, the possible spatio-temporal coincidence of β-gal-pH6 activity and cell proliferation in the developing olfactory epithelium was analyzed. Antibodies against PCNA and pHisH3 have been used to identify proliferative cells and mitotic figures in the postnatal mouse [27] and rat [47,48] olfactory epithelium. PCNA immunoreactive nuclei and pHisH3-positive mitotic figures were mainly located in the supporting cell and globose basal cell layers in 1-day-old mice (P1) (Figure 3A,B), and mainly in the globose basal cells in the 30-day-old (P30) (Figure 3C,D) and P60 mice (Figure 3E,F). Therefore, PCNA/pHisH3-immunoreactivity was always found basally or apically to the β-gal-pH6 staining band during the period analyzed.

**Figure 4:**
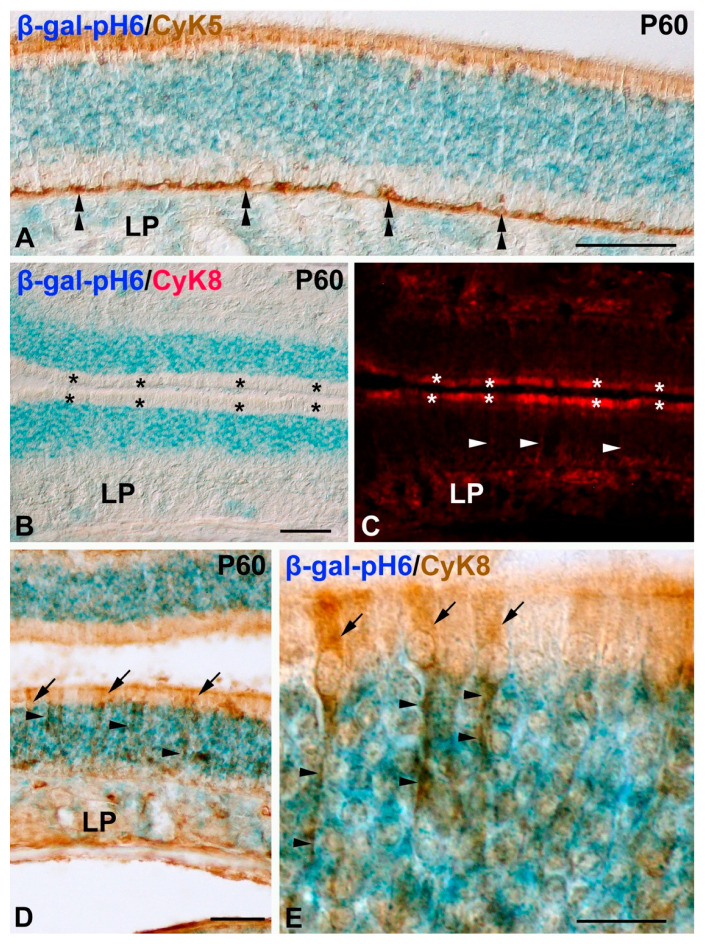
Relationship between β-gal-pH6 histochemical staining and markers of horizontal basal (**A**) and supporting cells (**B**–**E**) in the postnatal olfactory mouse mucosa. Cryosections of the P60 olfactory epithelium were doubly labeled with β-gal-pH6 histochemistry and antibodies against CyK5 (**A**) or CyK8 (**B**–**E**). (**A**) CyK5-immunoreactive horizontal basal cells were located in the basal surface of the olfactory epithelium (double arrowhead) in a region negative for the β-gal-pH6 staining. (**B**–**E**) CyK8 immunoreactive supporting cell bodies were always located apically to the β-gal-pH6 labeling (asterisks in (**B**,**C**); arrows in (**D**,**E**)). CyK8 immunoreactive fine processes from supporting cells were observed spanning the full extent of the epithelium (arrowheads in (**C**–**E**)). LP, lamina propria; OE, olfactory epithelium. Scale bars: 100 µm in (**A**–**D**); 15 µm in (**E**).

In order to identify horizontal basal cells and supporting cells in the developing mouse olfactory epithelium, we used antibodies against CyK5 [38] and CyK8 [34], respectively. CyK5 immunoreactive horizontal basal cells were clearly located in the basal region of the olfactory epithelium without any overlap with the β-gal-pH6 staining (Figure 4A). The cell somata of CyK8 immunoreactive supporting cells was always detected in the apical surface of the neuroepithelium and did not overlap with the dense band of β-gal-pH6 labeling (Figure 4B–E). Therefore, there is no chronotopographical coincidence between β-gal-pH6 staining and proliferating progenitors, including supporting and globose basal cells, CyK5-immunoreactive horizontal basal cells, and the cell somata of CyK8-immunoreactive supporting cells.

**Figure 5:**
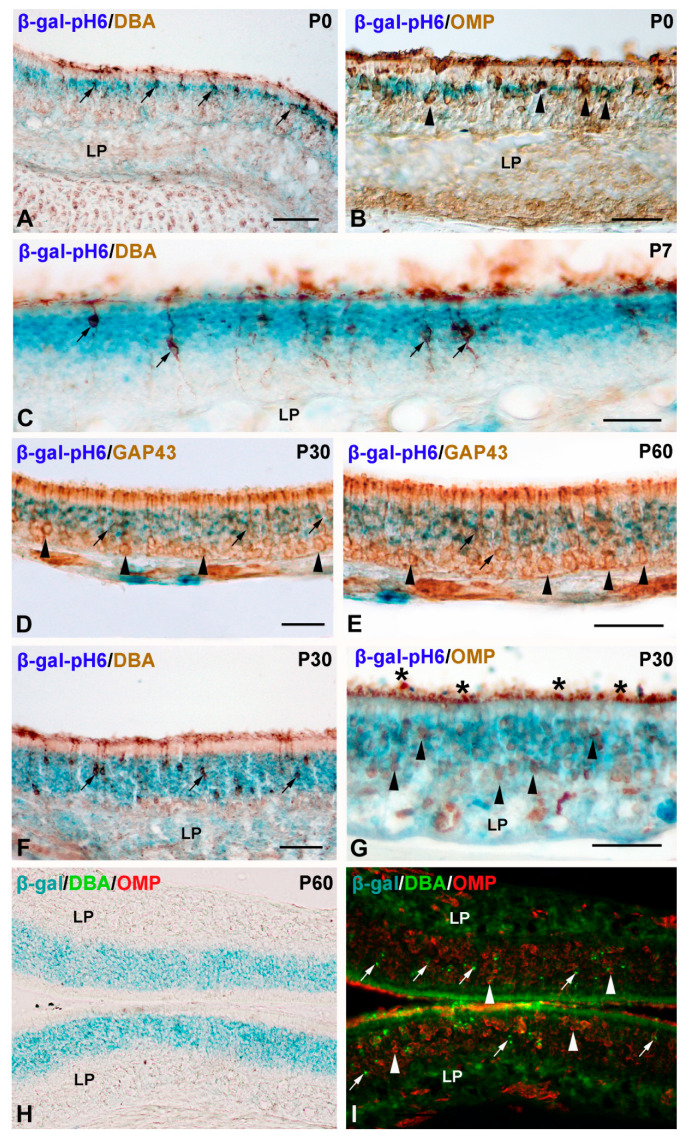
Relationship between β-gal-pH6 histochemical staining and neuronal markers in the developing olfactory mucosa in mice. (**A**,**B**) In the P0 olfactory epithelium, the cell somata of DBA-positive (arrows) or OMP-immunoreactive (arrowheads) neurons were always located inside the band of β-gal-pH6 staining. (**C**) In the P7 mouse olfactory epithelium, the cell somata of DBA-positive neurons coincided spatially with the β-gal-pH6 signal (arrows). At P30 (**D**) and P60 (**E**) most of the GAP43-immunoreactive neurons were located in the basal surface of the olfactory epithelium in a region negative for the β-gal-pH6 staining (arrowheads). Still, a few of them were found inside this band of labeling (arrows). At P30 (**F**,**G**) and P60 (**H**,**I**), the cell somata of DBA-positive (arrows) and OMP-positive (arrowheads) neurons were mainly found inside the band of β-gal-pH6 staining. Intesnse OMP-immunoreaction was detected in the apical surface of the epithelium (asterisks in (**G**)). LP, lamina propria; OE, olfactory epithelium. Scale bars: 100 µm.

These results suggested that olfactory neurons expressed high levels of β-gal-pH6. To test this hypothesis, we performed double-labeling experiments using a well-known neuronal marker of olfactory neurons in rodents, such as lectin histochemistry with DBA [44] or antibodies against OMP [33]. At P0, the cell somata of DBA-positive (Figure 5A) and OMP-positive (Figure 5B) olfactory neurons were arranged in a single row. These cell somata were always localized inside the thin β-gal-pH6 band of staining (Figure 5A,B). At P7, the cell somata of DBA-positive olfactory were distributed in different apicobasal positions, but they were always found inside the β-gal-pH6 band of labeling (Figure 5C). At P30 (Figure 5D) and P60 (Figure 5E) most of the GAP43-immunoreactive immature neurons were located basally to the β-gal-pH6 labeling, but a few of them were distributed inside the band of histochemical staining (Figure 5D,E). The same chronotopographical coincidence was found in the distribution of β-gal-pH6-histochemistry and the cell somata of DBA- and OMP-positive elements in the P30 (Figure 5F,G) and P60 (Figure 5H,I) mouse olfactory mucosa. All these data strongly suggest that β-gal-pH6 is highly expressed in differentiated neurons during postnatal development of the olfactory epithelium in the mouse. However, most GAP43 immunoreactive immature neurons are not labeled with this histochemical technique.

## 4. Discussion

The present study has provided the first description of the spatiotemporal location of β-gal-pH6 in the developing postnatal olfactory epithelium in vertebrates. Histochemical staining was always detected in a band of cells located in the intermediate region of the olfactory epithelium. The width of this band of staining increased with the advance of epithelial development, and its location coincided chronotopographically with the distribution of differentiated olfactory neurons in this tissue. However, GAP43 positive immature neurons located close to the olfactory epithelium’s basal lamina were negative for this histochemical staining. We also propose that this enzymatic activity is strongly enhanced in differentiated neurons in the developing olfactory epithelium but not in senescent cells.

The histochemical detection of β-gal-pH6 is a widely used biomarker of cellular senescence in both tissue sections and cultured cells [1,2]. More recently, it has been used to identify senescent cells in the developing vertebrate embryo [6,8,9,12,13,17,22,49,50,51,52,53]. In these studies, β-gal-pH6 staining closely correlated with the progression of ontogenetic cell death in structures such as the pronephros [52,53], the mesonephros [6,54], the heart [8], and the limb [7,9,12].

Cell death has been described as affecting olfactory neurons during early postnatal life in the mouse [27,55] and the rat [30], and even at adult stages [56]. However, apoptotic nuclei are scattered in the mouse olfactory epithelium during postnatal life [27]. Since β-gal-pH6 staining formed a dense, continuous band located in the intermediate layer of the olfactory epithelium, these results suggest no clear correlation between the pattern of β-gal-pH6 staining and the distribution of apoptotic figures in this tissue. Similar results have been described for other regions of the nervous tissue during development, such as the retina [23].

There was β-gal-pH6 activity detected in a band of cells located in the intermediate region of the olfactory epithelium. The width of this band and the intensity of histochemical staining increased progressively during the first days of postnatal life, coinciding chronotopographically with the distribution of cell somata of differentiated OMP-positive and DBA-positive olfactory neurons. A similar spatio-temporal pattern of neuronal differentiation has been described in the postnatal mouse [57] and rat [58] olfactory epithelium.

The β-gal-pH6 staining pattern suggests that this enzymatic activity is enhanced in the entire population of differentiated olfactory neurons. However, this staining is absent in immature neurons located in the basal region of the olfactory epithelium. Several studies have described intense β-gal-pH6 staining not only in neurons of the adult central nervous system [18,19,20,21,59,60,61,62] but even in subsets of neurons in very young mice [18,19,20,21]. These β-gal-pH6-labeled postmitotic neurons are also positive for other established markers of senescence, such as p16 or p21 [21,23]. More recently, strong β-gal-pH6 activity has been detected in subpopulations of early differentiated motoneurons in the rodent embryonic spinal cord [22]. Endogenous β-gal-pH6 activity has also been detected in the first differentiating retinal ganglion cells in the vertebrate retinal tissue [12,23], suggesting that this enzymatic activity is enhanced in some populations of neurons even at early stages of differentiation. Therefore, there are some similarities between detecting high levels of β-gal-pH6 in neurons of the olfactory epithelium and those of retinal tissue during embryonic development. However, in the olfactory epithelium of P60 mice, we found homogeneous β-gal-pH6 staining throughout the entire population of differentiated olfactory neurons. In contrast, this labeling in the differentiated retina is restricted to subsets of ganglion, amacrine, and horizontal cells [12,23,24].

## 5. Conclusions

Newborn cells located close to the basal surface of the olfactory epithelium undergo morphological changes as they differentiate into mature olfactory neurons. During this transition, they present an increase of endogenous β-gal-pH6 activity. These results are in concordance with previous studies describing this enzymatic activity in healthy subpopulations of neurons, even in embryonic tissues. Therefore, β-gal-pH6 histochemistry could be used as a histochemical and/or neuroanatomical tool because of its olfactory neuron-specific expression. Moreover, β-gal-pH6 activity has been demonstrated to be enhanced in other post-mitotic cells (skeletal muscle myofibers, choroid plexus, pancreatic cells, basal/stem cells in the intestinal epithelium, and osteocytes and osteoblasts) in the absence of disease or advanced aging and to co-localize with multiple senescence markers [16,21]. Some authors [15] suggest that this postmitotic cellular senescence observed in terminally differentiated cell types under physiological conditions could be implicated in tissue homeostasis. Therefore, some populations of post-mitotic cells are equipped with cellular machinery to engage programs involved in cellular senescence. This process could play a critical role in non-dividing cells during normal development or aging. Our study encourages exploring the possible function of β-gal-pH6 activity and other senescence markers in healthy tissues of embryonic, young, and middle-aged organisms.

## Data Availability

Some or all data used during the study are available from the corresponding author by request.
